# Analysis of Proteins and Piwi-Interacting RNA Cargo of Extracellular Vesicles (EVs) Isolated from Human Nose Organoids and Nasopharyngeal Secretions of Children with RSV Infections

**DOI:** 10.3390/v17060764

**Published:** 2025-05-28

**Authors:** Tiziana Corsello, Nicholas Dillman, Yingxin Zhao, Teodora Ivanciuc, Tianshuang Liu, Antonella Casola, Roberto P. Garofalo

**Affiliations:** 1 Department of Pediatrics and Center for Lung Disease Inflammation and Remodeling (LUDIR), The University of Texas Medical Branch at Galveston (UTMB), Galveston, TX 77555, USA; ancasola@utmb.edu (A.C.); rpgarofa@utmb.edu (R.P.G.); 2 Department of Pediatrics, The University of Texas Medical Branch at Galveston (UTMB), Galveston, TX 77555, USA; 3 Department of Internal Medicine, University of Texas Medical Branch, Galveston, TX 77555, USA; yizhao@utmb.edu

**Keywords:** RSV, extracellular vesicles, nasal secretion, nose organoid, children

## Abstract

Respiratory syncytial virus (RSV) is the leading cause of respiratory infections in children. Extracellular vesicles (EVs), released by airway epithelial cells, contain proteins and different families of non-coding RNAs (EV cargo) that can modulate the responses of target cells to viral infection. Nasal mucosa is a primary site of viral entry and the source of EVs present in the upper airway secretions. In this study we characterized proteins, including inflammatory mediators and cytokines, and the piwi-interacting RNA (piRNAs) cargo of EVs isolated from pediatric human nose organoids (HNO) and nasopharyngeal secretions (NPS) positive for RSV. Using Proximity Extension Assay (PEA) and Luminex multi-target arrays, we found significant enrichment in several chemokines and other mediators/biomarkers, including CCL2, CCL20, CXCL5, CX3CL1, CXCL6, MMP-1, MMP-10, uPA, Flt3L, ARNT and CD40 in EVs secreted by RSV-infected HNO compared to control mock HNO. Analysis of NPS samples from RSV infected children revealed that CCL3, CCL20, CXCL8, uPA, VEGFA, were concentrated in the NPS-EV fraction. LC-MS/MS and Gene Ontology indicated that RSV positive NPS-EVs originate from different cellular sources, with the most abundant proteins from neutrophils and epithelial cells. A total of 490 piRNAs were detected by NGS sequencing of small RNA libraries obtained from NPS-EVs, which has not been reported prior to this study. Identification of inflammatory mediators and small non-coding RNAs which are compartmentalized in EVs contributes to understanding mechanisms of virus-mediated pathogenesis in RSV infections.

## 1. Introduction

Respiratory syncytial virus (RSV) is the largest contributor to acute respiratory infections in children and is responsible for 3.6 million hospital admissions worldwide [1]. Along with the hospitalizations, RSV is also responsible for over 100,000 deaths with most deaths coming from low- or middle-income countries [1]. In addition to acute morbidity, RSV infections during the first two years of life have been linked to the subsequent development recurrent episodes of wheezing, allergic sensitization, development of asthma, and persistently reduced lung function [2,3,4,5,6]. RSV vaccines for the pediatric population are not yet available, with prevention of severe infections limited to the first months of life by passive immunization of neonates/young infants with monoclonal antibodies (Nirsevimab) or prenatal transfer of neutralizing antibodies by maternal immunization in pregnancy [7,8]. Specific therapeutic interventions for RSV infections are not yet available, thus studies improving the understanding of the disease pathogenesis are warrant.

Cytokine-driven inflammation as well as functional immaturity of the immune system, in particular reduced functionality of interferon (IFN) pathways during the early period of life, have been considered among the mechanisms involved in the severity of viral respiratory infections, including RSV-mediated infection [9,10]. Extracellular vesicles (EVs) are nanoparticle-sized released from cells into the extracellular environment and delimited by a lipid bilayer carrying nucleic acid, lipid, and protein cargo. EVs transfer their biologically active cargo between neighboring cells and to distant sites, contributing to cell–cell communication, inflammation, and disease pathogenesis [11]. EVs are surrounded by a lipid bilayer, which protects their molecular cargo from extracellular degradation, facilitating cytokine delivery and targeting of distant cells [12]. This structure also makes EVs highly stable in storage [13] and available in biofluids such as blood, urine, saliva, and nasal lavage [14,15,16,17]. Since EVs carry a diverse content of biological materials, including proteins and small noncoding RNAs (sncRNAs), modifying EV cargo offers a promising strategy for delivering drugs and specific biomolecules to target cells. Piwi-interacting RNAs (piRNAs), of about 26–32 nucleotides in length, are the largest class of sncRNA molecules expressed in animal cells. piRNAs were first identified in germ cells [18], and they play a crucial role to safeguard the genome by maintaining genome complexity and integrity [19]. More recently, they have been detected in various organs and somatic cells [20,21], with their distinct expression patterns linked to the progression of numerous diseases, such as cancer [22], diabetes [23], and cardiovascular [24] conditions. We have published that RSV infection significantly changes the piRNA profile in normal human small airway epithelial cells (SAEC) [25], and piRNAs are a major component of the sncRNA cargo of EVs secreted by A549 cells infected with RSV [26]. Despite these advancements, our understanding of protein and piRNA expression changes and their potential roles during viral infections remain limited.

Based on our previous studies in lower airway epithelial cell-derived EVs following RSV infection, this study was designated to analyze pro-inflammatory, immunomodulatory proteins and piRNAs within EVs from the upper airway in response to RSV infection, highlighting the role of EV cargo in mediating disease via cellular crosstalk from upper to lower airways. Notably, the structural integrity of EVs secreted by the upper airway mucosa—an entry point for respiratory viral pathogens—safeguards their protein and RNA cargo from degradation, even in inflamed airways. Using the in vitro and ex vivo models, we found significant enrichment of cytokines and chemokines in EVs from pediatric human nose organoids (HNO) 3D cultures and nasopharyngeal secretions (NPS) of RSV-positive children. Additionally, piRNAs were detected at the EV level of NPS samples of children with episodes of RSV infection using next generation sequencing (NGS) analysis. These findings provide insights into protein and piRNA cargo of upper airway derived EVs, enhancing insight into virus-mediated mechanisms of disease in young RSV patients. The packaging of innate immune mediators, such as cytokines, in EVs could protect them from cellular degradation and be indeed a crucial mechanism for regulating innate and antiviral responses both locally and distantly from the initial site of viral entry, which might otherwise evade targeting by cytokines in their soluble, circulating form.

## 2. Materials and Methods

### 2.1. Human Nose Organoid (HNO) Air-Liquid Interface (ALI) Culture and RSV Infection

Pediatric human nose organoid (HNO) in air-liquid interface (ALI) culture were generated and cultured by the Baylor College of Medicine (BCM) 3D Organoid Core as published in [27,28]. Briefly, nasal washes and swabs were collected from infants (<2 years old) under IRB-approved protocols at BCM and used to establish human nasal organoids (HNOs) [28]. Following enzymatic digestion and debris removal of nasal samples, cells were embedded in Matrigel^®^ and cultured in growth media to allow 3D organoid expansion for 3–4 days. The mature 3D HNOs were enzymatically and mechanically sheared to make a 24-well plate format ALI culture. Clear 24-well plate Transwells were precoated with 100 μL of bovine type I collagen at 30 μg/mL. HNOs were dissociated using 0.5 mM EDTA. Single cells were obtained by adding 0.05% trypsin/0.5 mM EDTA and pelleted at 400× *g* at room temperature for 5 min to generate single cells. The pellet was resuspended in a modified StemCell PneumaCult™-Ex Plus medium, supplemented with 10 μM Y-27632, a ROCK inhibitor commonly used to improve the efficiency and survival of nasal organoids, plus the epidermal growth factor (EGF). The single cells were added at a seeding density of 3 × 10^5^ cells/well. After 4 days, confluent monolayers were cultured using a differentiation medium (PneumaCult-ALI medium from STEMCELL Technologies) in the lower compartments of the Transwells until 21 days. Pediatric HNO-ALI 9003 line was used for this study representing the human nose organoid line and the number gave of the participant (number 9003) from which it was obtained. RSV stocks and viral pools were prepared as previously described [26]. When the differentiated HNO protocol was completed, HNO-ALI were subsequently infected with sucrose-purified RSV (Long strain) at a multiplicity of infection (MOI) of 1 (30 μL/well) at the apical side of the culture. MOI of 1 was chosen based on our prior publications [26,29,30] and preliminary experiments indicating that these doses provide efficient and physiologically relevant infection in airway epithelial cells, without causing excessive cytotoxicity or overwhelming antiviral responses that may mask treatment effects. An equivalent amount of 30% sucrose solution was added to uninfected HNO-ALI cells as mock control.

### 2.2. Collection of the Nasopharyngeal Secretion (NPS) Samples

NPS samples were collected as part of an ongoing IRB-approved study on the pathogenesis of lower respiratory tract infections in children younger than 2 years of age. After written informed consent was provided by the parent or legal guardian of hospitalized children, NPS samples were collected within the first 24 h after hospital admission, as previously described [31,32,33,34,35,36,37,38]. Control NPS were children admitted to the Pediatric Intensive Care Unit following surgery for conditions unrelated to airways disease and negative for viral infections. Samples were immediately transported to the laboratory on ice, aliquoted and stored at −80 °C. Following thawing, one aliquot of the NPS sample was tested for respiratory viruses, using the multiplex RT-PCR-based Luminex xTAG Respiratory Viral Panel (RVP, Luminex Molecular Diagnostics to detect simultaneously 19 viral targets; one aliquot was used for direct analysis of inflammatory proteins in the NPS fluid, and another aliquot for the isolation of extracellular vesicles (EVs).

### 2.3. Isolation and Purification of Extracellular Vesicles (EVs) from HNO-ALI Culture Supernatant and from NPS Samples

EVs were isolated from HNO-ALI supernatant and from sixteen NPS samples according to our two-step immunopurifcation protocal as previosly described [26,38]. Since RSV is predominantly released from the apical site of HNO-ALI [39], we purified EVs from 2 mL of apical culture media collected at 24 h post-infection. Prior to EV isolation procedure, NPS samples were treated with 5% Sputolysin, vortexed for 30 s, and left at room temperature for 15 min to dissolve the mucus. HNO-ALI supernatant and mucous-free NPS samples (1 mL) were subjected to debris removal by centrifugation at 3000× *g* for 15 min at 4 °C and then subjected to further debris cleaning by filtration through 0.22 μm sterile filters. Exoquick-TC (System Biosciences, Palo Alto, CA, USA) reagent was added to samples, mixed thoroughly, and incubated overnight at 4 °C to precipitate EVs. Next morning the mixture was subjected to centrifugation at 1500× *g* for 30 min, the EV pellets were washed and resuspended in filtered PBS. EVs were subjected to CD63 immuno-purification using CD63 exosome isolation reagents (System Biosciences, USA), following manufacturer’s instructions. The purified EVs were eluted from the bound CD63 beads in an average of 300 μL and used for experimental procedures.

### 2.4. EV Size and Particle Number Measurements

EVs size distribution and number of particles were analyzed using a ZetaView PMX 110 Tracking Analyzer (Particle Metrix GmbH, Meerbusch, Germany) and software (Zeta-View^®^ 8.04.02, Particle Metrix GmbH). EV samples were measured three times to ensure reproducibility. The analyzer was cleaned between samples using filtered water.

### 2.5. Exosome Antibody Arrays (Exo-Check)

Protein concentration of EV preparations was determined using a protein assay (Bio-Rad) and 10 μg resuspended in 1X PBS and mixed with lysis buffer to achieve a final concentration of 10% lysis buffer. Then 1 μL of labeling reagent was added to the sample and vortexed. The sample was then incubated for 30 min at room temperature. A column filter was used to remove the excess labeling reagent. The labeled EV lysates were combined with 5 mL of blocking buffer and poured over a membrane to fully submerge the membrane in liquid. The membrane was incubated overnight at 4 °C on a shaker. The membrane was then washed, and 5 mL of detection buffer was added to the membrane. The membrane then incubated for 30 min at room temperature on a shaker. After washing the membrane again, EV protein markers were detected using enhanced chemiluminescence (ECL). The primary antibodies used with the Exo-Check EV-specific array (System Biosciences, USA) are eight: CD63, CD81, ALIX, FLOT1, ICAM1, EpCam, ANXA5 and TSG101. The cis-Golgi marker (GM130) monitors for other cellular compartment contamination.

### 2.6. Proximity Extension Assay (PEA) and Multiplex Cytokine Analysis

EV isolated from HNO-ALI and NPS and NPS biofluids were tested for inflammatory biomarkers, including cytokines/chemokines using multiplex assays. EV samples quantified by ZetaView PMX 110 tracking analyzer were normalized to the same particle number and protein concentrations prior to the assays. Proximity Extension Assay (PEA) was performed by the Olink Target 92-target inflammation panel (Olink Bioscience AB, Uppsala, Sweden). The PEA technology is based on two paired oligonucleotide antibodies, referred to as probes, which bind to the target protein. When the DNA of the two antibodies come in proximity to each other, the DNA hybridized. DNA polymerase then extended the oligonucleotides which was quantified by quantitative PCR using a log 2 scale that reports the data in an arbitrary unit of NPX (normalized protein expression levels). The higher the NPX, the higher the protein concentration. Some NPX values in certain groups were below the limit of detection (LOD), but based on Olink’s guidelines, all NPX data were used to identify statistically significant differences between groups as using data below the LOD typically doesn’t increase false positives in statistical tests. Samples that failed technical criteria were excluded. Cytokines and chemokines were measured as pg/mL using the 45-target human multi-Plex panel (Bio-Rad Laboratories, Hercules, CA, USA) according to the manufacturer’s instructions.

### 2.7. Liquid Chromatography–Tandem MS (LC-MS/MS) Analysis of NPS Derived EVs and Data Processing

Briefly, ~10 μg of each EV sample were cleaned and trypsin-digested using suspension traps, and the resulting peptides were analyzed with a nanoflow LC-MS/MS chromatography system (UltiMate 3000 RSLCnano, Dionex, Waltham, MA, USA) coupled to a Thermo Orbitrap Eclipse mass spectrometer (Thermo Fisher Scientific, San Jose, CA, USA) as described previously [40,41,42]. The MS data were analyzed using MaxQuant software version 1.5.2.8 and the Andromeda search engine [43,44]. The required false positive rate for identification was set to 1% at the peptide level and 1% at the protein level. Quantification in MaxQuant was performed using the built-in XIC-based label-free quantification (LFQ) algorithm. The LFQ values were log2-transformed, and a Student’s *t*-test was used to assess the statistical significance of protein abundances. Genome ontology (GO) analysis of proteins significantly elevated in the NSP EVs of RSV-infected compared to uninfected patients was performed with Panther platform [45] (http://pantherdb.org/).

### 2.8. RNA Extraction of NPS-EVs and Next Generation Sequencing (NGS)

RNA was extracted from NPS-derived EVs using the SeraMir EV RNA purification column kit (System Biosciences, USA) according to the manufacturer’s instructions and RNA was sent to the Genomic and RNA profiling Core (GARP) at BCM for libraries preparation and sequencing. Small RNA libraries were made using the QIAseq^®^ miRNA Library Kit (QIAGEN). cDNA library was constructed, size selected and analyzed for quality with Bioanalyzer. EVs NGS were performed on an Illumina NextSeq550 (single end 75 base) using TruSeq SBS kit v3 (Illumina) and protocols defined by the manufacturer and published [46]. Sequencing data were analyzed to identify piRNAs and differences in expression between infected and uninfected control children.

### 2.9. Bioinformatics and Statistical Analysis of piRNA Data

piRNA expression was quantified using piRNAdb and featureCounts, with normalized read counts used for analysis. Differential expression of piRNAs was conducted in R using EdgeR. Features with >1.5-fold change and FDR with a *p* value < 0.05 underwent for functional analysis. piRNA targets were identified using piRNAQuest [47], UCSC liftOver [48], and BEDTools [49].

### 2.10. Statistical Analysis

Statistical analysis of size and particle measurement, NPX and Bioplex concentrations data comparing EV groups (HNO-ALI EVs of RSV vs. Control; NPS EVs vs. NPS biofluids), was performed using a paired student’s *t*-test (GraphPad Prism 9.5.1; GraphPad Software, Inc., San Diego, CA, USA). Results are expressed as mean ± SEM for each experimental group. Significance was considered when *p* < 0.05 (*) or *p* < 0.01 (**). Raw normalized protein expression (NPX) data from the Olink platform were transformed by scaling, yielding values with a mean of 0 and standard deviation of 1.

## 3. Results

### 3.1. Characterization of HNO-Derived EVs

**Figure 1:**
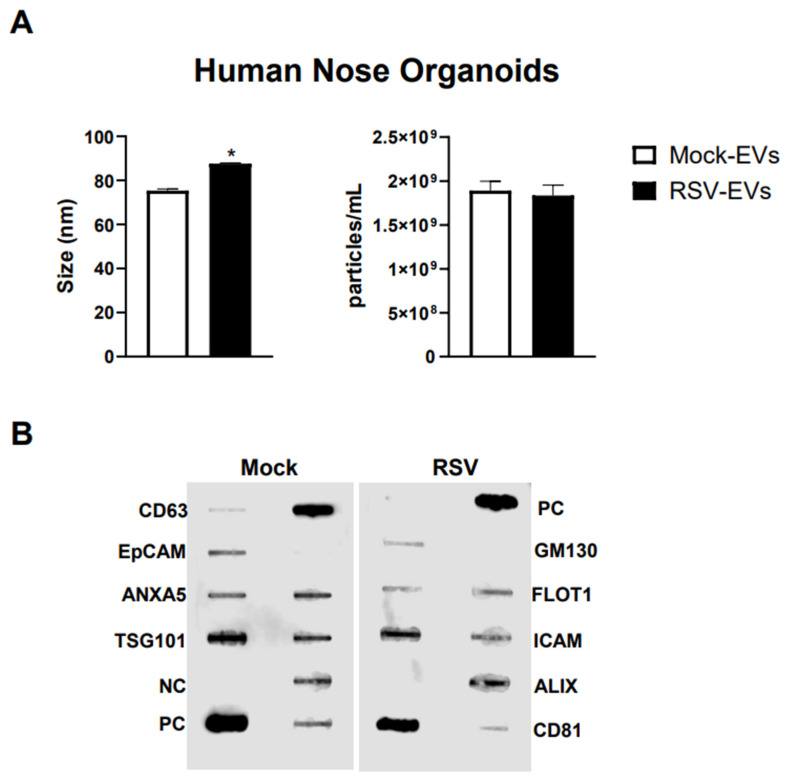
Characterization of EVs from human nasal organoids in response to RSV infection. Absolute size and concentration were determined by Zetaview analysis of EVs isolated from (**A**) mock (uninfected) (open bars) and RSV-infected (black bars) human nasal organoids. * indicates a statistical difference comparing RSV- EVs versus Mock-EVs (* *p* value < 0.01). Data is representative of three independent experiments. (**B**) Exocheck array analysis showing the detection of eight known EV marker proteins in human nose organoids derived EVs using 10 µg of total protein lysates. PC = positive control; NC = negative control; EV markers: CD63, Epithelial cell adhesion molecule (EpCam), Annexin A5 (ANXA5), Tumor susceptibility gene 101 (TSG101), Flotillin 1 (FLOT1), Intercellular adhesion molecule 1 (ICAM1), Programmed cell death 6 interacting protein (ALIX) and CD81. Cis-Golgi matrix protein marker (GM130) serves as control to monitor cellular contamination in EV preparations.

We isolated EVs from RSV-infected or mock inoculated pediatric HNO-ALI cultures using a two-step method of precipitation followed by CD63 immuno-beads purification. We have previously shown that this protocol eliminates contamination of EVs by replicating virus, as we confirmed also in representative preparations of HNO-ALI EVs [26]. As from per the guidelines of the International Society of Extracellular Vesicles, we measured EV size distribution, number of particles, and expression of EV markers to confirm the purity and identification of the EV samples [50]. We found that the average size of EVs from uninfected (mock-EVs) and RSV infected HNO cells (RSV-EVs) were 75.3 nm and 87.6 nm, respectively (Figure 1A). Mock HNO produced an average of 1.89 × 10^9^ particles/mL, and RSV-infected HNO produced an average of 1.83 × 10^9^ particles/mL (Figure 1A). We confirmed by a membrane-based Western blot multiple target assay that EVs isolated from HNO cells expressed the typical EV markers CD63, CD81, ICAM, EpCAM, ANXA5, FLOT1, TSG101 and ALIX. EVs were negative for the cis-Golgi matrix protein GM130 (Figure 1B), a non-EV marker, suggesting we successfully isolated pure EVs. Of interest, we observed an increase in ALIX expression by RSV-EVs compared to mock-EVs and a slightly lower expression of EpCAM and CD81 markers on EVs from RSV-infected vs. mock-EVs.

### 3.2. Analysis Inflammatory Proteins Associated with of HNO EVs by Proximity Extension Assay (PEA) and Bioplex Arrays

To broaden the characterization of EVs from the human upper airway in response to a viral infection, we used Olink technology along with the Bioplex platform to profile inflammatory protein associated with RSV-infected or mock control HNO-secreted EVs. The Olink platform uses the multiplex Proximity Extension Assay (PEA) technology to analyze protein biomarkers involved in key biological processes such as adaptive immune response, defense response to virus, lymphocyte activation, inflammatory response and cytokine-mediated signaling pathways. The suggested protein concentration for Olink analysis was between 0.5 and 1 mg/mL. Samples were run undiluted and standardized to the same particles number across all samples. Data are presented in log2-scale as Normalized Protein eXpression (NPX).

**Figure 2:**
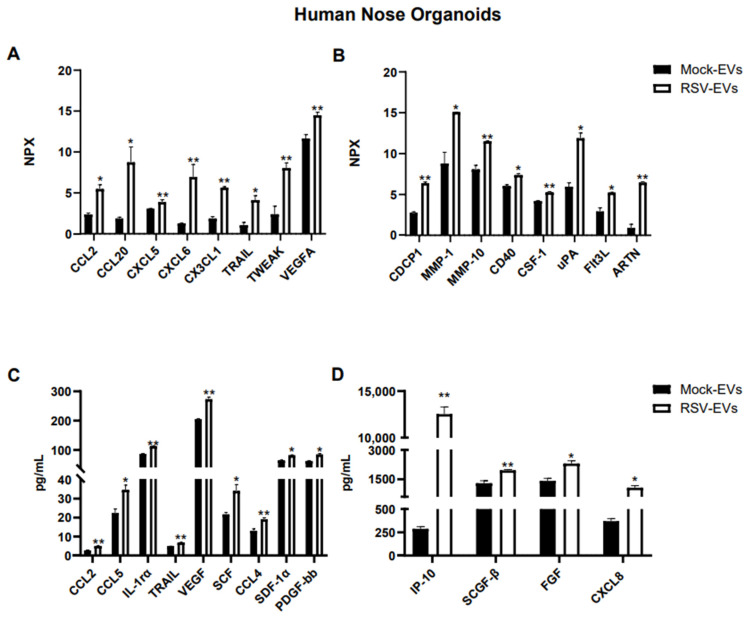
Protein cargo profiling of EVs isolated from RSV-infected and uninfected (Mock) HNO cultures using (**A**,**B**) Proximity Extension Analysis (PEA) and (**C**,**D**) Bioplex analysis. Data are presented as mean ± SEM. * and ** indicates a statistical difference comparing RSV- EVs versus Mock-EVs (* *p*-value < 0.05; ** *p*-value < 0.01).

Out of ninety-two proteins included in the Olink human inflammatory panel, fifty-one proteins were detected within the detectable range in EVs isolated from HNO cultures. As shown in Figure 2A,B, sixteen immune proteins were significantly enriched in EVs from RSV infected HNO compared to mock-HNO: the chemokines CCL2, CCL20, CXCL5, CXCL6, and CX3CL1, TNF-related apoptosis-inducing ligand (TRAIL), TNF-related weak inducer of apoptosis (TWEAK), the vascular endothelial growth factor A (VEGF-A), the glycoprotein CUB domain-containing protein 1 (CDCP1), the matrix metalloproteinases 1 and 10 (MMP-1, MMP-10), the costimulatory protein CD40, Colony Stimulating Factor 1 (CSF-1), the urokinase-type plasminogen-activator (uPA), Fms-like tyrosine kinase 3 ligand (FLT3L), and the cytokine Artemin (ARTN).

Additional analysis of EV-associated proteins was performed by the Bioplex platform. Using the human 48-human multi-Plex array (Bio-Rad Laboratories), we found forty-six proteins which were detected in Mock- and RSV-EVs from HNOs within the standard range concentration. Similar to the finding in the Olink platform CCL2, TRAIL and VEGF were significantly increased in RSV-EVs compared to Mock-EVs, along with IL-1rα, CXCL8, IP-10, CCL4, CCL5, SCF, SCGF-β, SDF-1α and FGF (Figure 2B,C).

### 3.3. Purification and Characterization of EVs from Nasopharyngeal Secretion (NPS) of Children Positive for RSV

**Figure 3:**
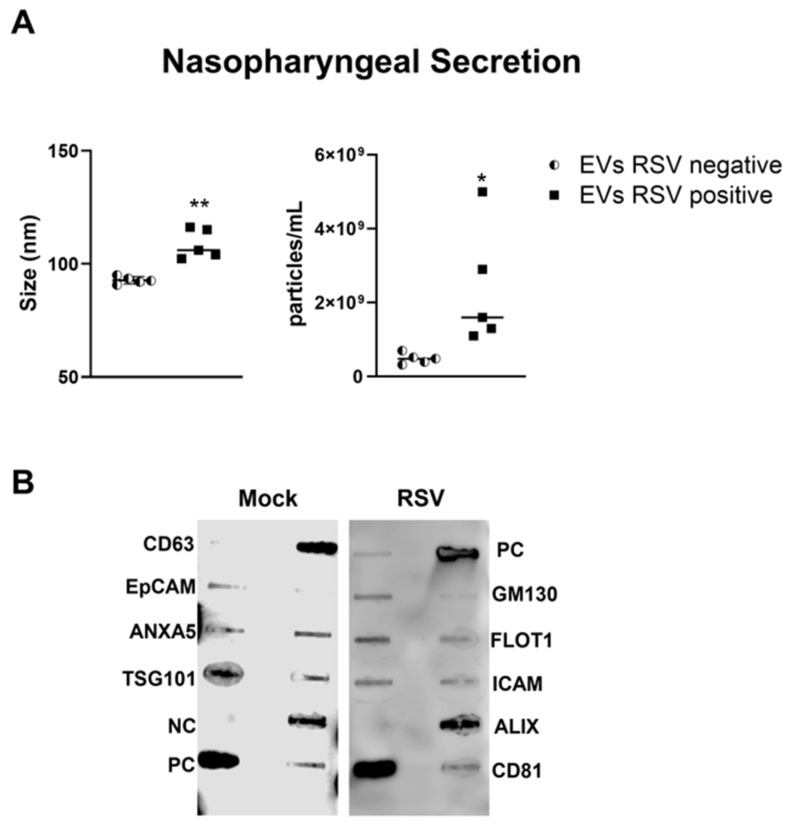
Characterization of nasopharyngeal secretion-derived EVs from children with (positive) and without (negative) RSV infection. (**A**) Absolute size and concentration (right) were determined by Zetaview analysis of EVs isolated from RSV-negative and RSV-positive nasopharyngeal secretion. * and ** indicates a statistical difference comparing RSV- positive versus RSV- negative (* *p*-value < 0.05; ** *p*-value < 0.01). (**B**) Exocheck array analysis showing the detection of eight known EV marker proteins in nasopharyngeal secretion derived EVs using 10 µg of total protein lysates.

We collected nasopharyngeal samples (NPS) from sixteen children up to 2 years of age who were hospitalized for respiratory tract infections. Five NPS samples were obtained from uninfected (control) children. The presence of RSV was confirmed using the Luminex xTAG Respiratory Viral Panel. EVs were isolated from NPS samples using a two-step purification method as previously described [26]. We found that the size of the nanoparticles in RSV-EVs derived from NPS was 104 nm, whereas in control-EVs (RSV negative) was 92.7 nm (Figure 3A). The mean concentration of nanoparticles in the EVs derived from RSV positive NPS (RSV-EVs) samples was 1.13 × 10^9^ particles/mL, while in EVs derived from NPS negative for RSV (control-EVs) was 4.84 × 10^8^ particles/mL (Figure 3B). We confirmed the presence of EV markers in NPS-derived EVs using the exosome antibody array and found EVs to be negative for the cis-Golgi matrix protein GM130 (Figure 3B).

### 3.4. Analysis of EV Protein Cargo for Inflammatory Biomarkers in NPS and NPS-Derived EVs by Proximity Extension Assay (PEA) and Bioplex Platform

**Figure 4:**
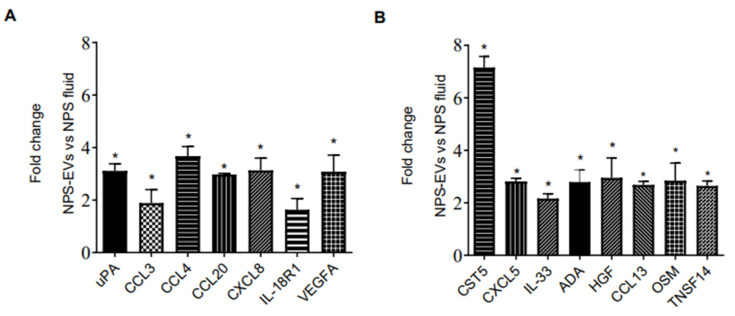
(**A**,**B**) Proximity Extension Analysis (PEA) (**B**) human multi-plex Bioplex panel array of NPS samples and NPS-derived EVs from children with RSV infection. Data are presented as mean ± SEM. Statistically significant enriched inflammatory proteins (* *p*-value < 0.05) comparing RSV-EVs versus RSV-positive NPS samples.

We then investigated whether the protein profile observed in EVs isolated from 3D nose organoid cultures were representative of that in EVs from nasal biofluid using PEA. We profiled the inflammatory proteins in NPS biofluid positive for RSV as well as in EVs derived from the same NPS sample. Surprisingly, we observed that most proteins were present at higher levels in EVs isolated from RSV-positive NPS compared to the RSV-positive NPS biofluid. As expected, EVs from RSV-positive NPS had the highest concentrations of fifty out of the ninety-two proteins tested. Fifteen proteins were expressed at significantly higher levels in RSV-positive EVs compared to NPS biofluid: uPA, CCL3, CCL4, CCL20, CXCL8, IL-18R1, VEGFA, Cystatin D (CST5), CXCL5, IL-33, ADA, HGF, CCL13, Oncostatin-M (OSM) and TNFSF14, Figure 4A,B. Notably, CST5, CXCL8 and VEGFA displayed higher levels in EVs than in nasal biofluid.

**Figure 5:**
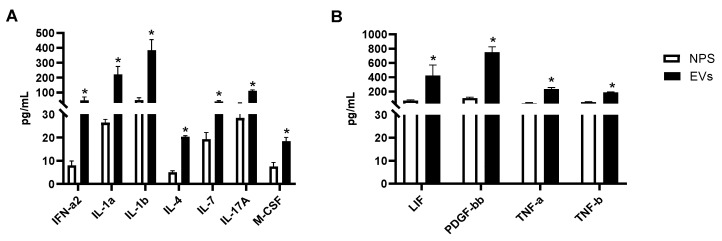
(**A**,**B**) Human multi-plex Bioplex panel array of NPS samples and NPS-derived EVs from children with RSV infection. Data are presented as mean ± SEM. Statistically significant enriched inflammatory proteins (* *p*-value < 0.05) comparing RSV-EVs versus RSV-positive NPS samples.

We then investigated whether other cytokines and chemokines were present in EVs or NPS samples. Forty-six proteins were detected within the detectable range in EVs isolated from NPS as well as in NPS biofluid using Bioplex. A comparison of inflammatory proteins between RSV-EVs and RSV-positive NPS biofluid showed significantly higher concentrations of eleven proteins in RSV-EVs isolated from nasal biofluid compared to RSV-positive NPS, shown in Figure 5A,B.

### 3.5. Analysis of EV Protein Cargo in NPS-Derived EVs by LC-MS/MS

**Figure 6:**
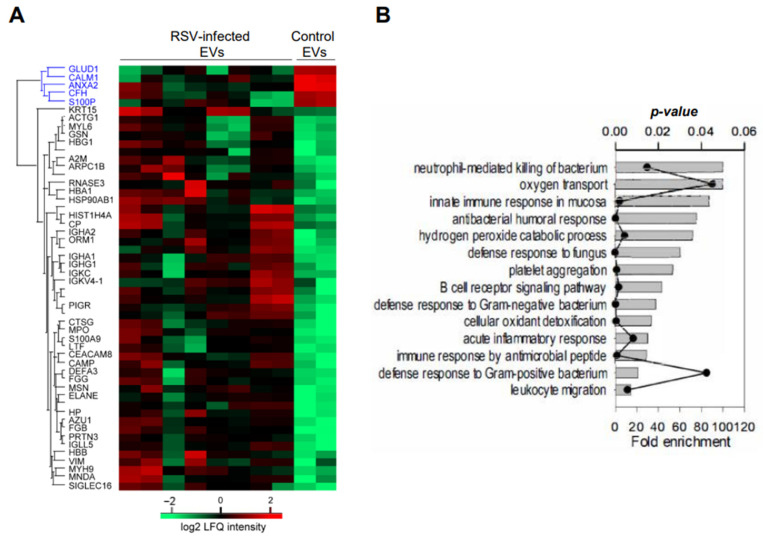
(**A**) LC-MS/MS of four RSV-infected and one control uninfected NPS-EV samples. Unsupervised hierarchical clustering of significant proteins. Heatmap was based on Z-score-normalized log2 protein expression. (**B**) GO biological processes enrichment analysis for the proteins which were up-regulated by RSV infection (*p*-value < 0.05 with Bonferroni correction for multiple testing). Each annotation is displayed by fold enrichment (bar) and *p*-value (scatter plot).

We have performed initial mass spectrometry studies of NPS-EVs from four infant patients with RSV bronchiolitis and one age-matched uninfected controls, each run in duplicate. We found fifty-two significant proteins including five downregulated proteins: Glutamate dehydrogenase 1 (GLUD-1), Calmodulin (CALM1), Annexin A2 (ANXA2), Complement factor H (CFH) and Protein S100-P (S100P), and forty-seven upregulated in NPS derived EVs from RSV infected compared to control children (*p* ≤ 0.05) as shown in Figure 6A.

We examined the abundance of cellular markers of B cells, epithelium cells, eosinophils, and neutrophils using intensity-Based Absolute Quantification and found that NPS- derived EVs came from several cellular sources, with the most abundant proteins originating from neutrophils (Table 1).

Additionally, based on the log2 fold changes, RSV induced a strong release of NPS-EVs from neutrophils and eosinophils, compared to epithelial cells. GO biological processes enrichment analysis for the upregulated proteins are presented in Figure 5B, which shows strong enrichment of GO biological function terms associated with innate immune response in airway mucosa.

### 3.6. Analysis of piRNA Cargo in NPS-Derived EVs by NGS Analysis

**Figure 7:**
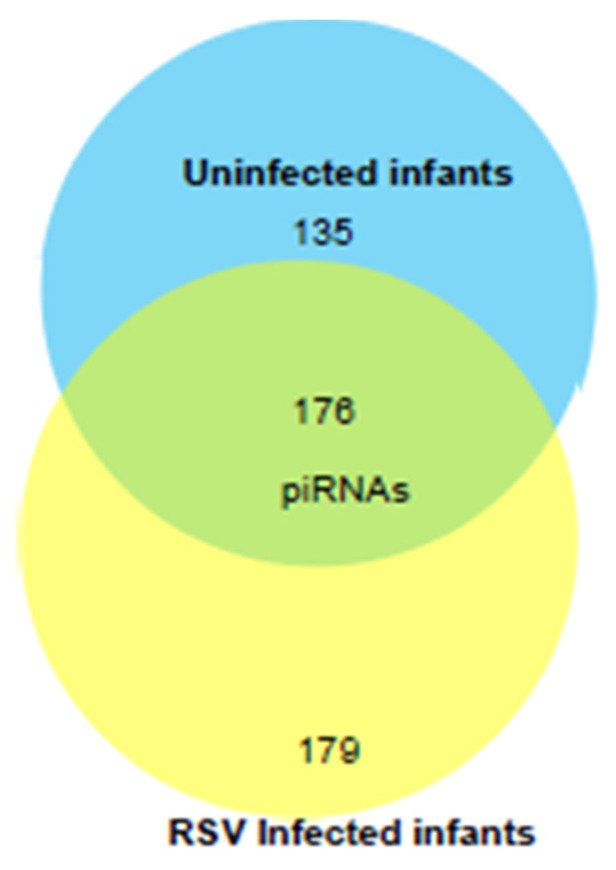
Venn diagram of the total number of piRNAs (after removing low abundance ones) detected by NGS in EVs from NPS of ten RSV-infected and four uninfected children. Number in the overlapping area represents the detected piRNAs shared between the two groups.

We next profiled the piRNA composition in a pilot study of EVs isolated from six samples of RSV-positive children and three uninfected (control) using NGS analysis. piRNAs are a class of snRNAs (26–32 nucleotide long) also involved in epigenetic and post-transcriptional gene silencing mechanisms [54,55]. A total of 490 piRNAs were detected in EVs from NPS samples of uninfected and RSV-infected infants. Of these, one hundred seventy-six piRNAs were detected in both groups. One hundred seventy-nine piRNAs were present only in the RSV group, and one hundred thirty-five piRNAs were present only in the uninfected group as represented in the intersecting Venn diagram, Figure 7.

We found that four piRNAs were significantly upregulated, piR-32956, piR-33036, piR-33005 and piR-14633, and one downregulated piR-33149 in EVs isolated from RSV-infected samples compared to uninfected group. Potential targets of the significantly up- and down-regulated piRNAs following RSV infection are listed in Table 2. Most of the predicted targets belong to the class of miscellaneous RNA (miscRNAs) and long noncoding RNAs (lncRNAs), including Y RNA and long intergenic non-coding RNAs (lincRNAs) such as LINC00623.Y RNA belong to the small non-coding RNA family participating in DNA replication, RNA quality control and cellular stress responses [56]. LincRNAs are autonomously transcribed RNAs longer than 200 nucleotides in length that do not overlap protein-coding genes [57].

## 4. Discussion

The main goal of this study was to identify protein mediators associated with EVs released by the upper airway mucosa, a subcellular pathway involved in cell–cell communication that influences biological responses in distant tissues, such as the lower airways. EVs could be exploited as biomarkers of disease severity and a therapeutic approach for respiratory syncytial virus (RSV).

RSV is the leading cause of respiratory tract infections in children worldwide, annually resulting in 33.1 million cases and 3.2 million hospitalizations in children < 5 years old [58]. Nowadays, treatments in infants against RSV infection are mainly supportive and limited to passive immunization using monoclonal antibodies [59]. RSV vaccines for the pediatric population are currently not available, and some of the critical molecular mechanisms driving virus-induced respiratory tract disease remain poorly understood [60].

The nasal epithelium is the first entry site and line of defense against RSV and thus can drive inflammatory and antiviral responses in the lower airways. Virus-infected respiratory epithelial cells produce innate immune mediators such as cytokines and interferons (IFNs) that can be detected in biofluid such as airway secretions of infected patients [61]. EVs are nanoparticle-sized released from cells into the extracellular environment and delimited by a lipid bilayer carrying nucleic acid, lipid, and protein cargo. EVs transfer their biologically active cargo between neighboring cells and to distant sites, contributing to cell–cell communication, inflammation, and disease pathogenesis [11,62]. EVs are surrounded by a lipid bilayer, which protects their molecular cargo from extracellular degradation, facilitating cytokine delivery and targeting of distant cells [12]. Various studies have reported that cytokines can be concentrated within EVs [12,63,64] and exert their activity at the surface of other cells that might not otherwise be targeted by cytokines in soluble, circulating form [65]. While in vitro studies show that EVs may promote pathogen transmission and spreading of viral infection (e.g., HCV, HIV) [66,67,68,69], other studies show that EVs can limit viral spread (e.g., Dengue) [70]. We recently observed, unexpectedly, that human primary lower epithelial cells contain measurable levels of both type I and type III IFNs. These EV-associated IFNs were biologically active and markedly reduced viral replication in recipient cells [38]. Currently, the role of EVs from upper airway shaping the lower airway response in viral infections is unexplored.

To our knowledge, we are the first group to isolate and purify EVs from pediatric upper airway infected with RSV using both in vitro and ex vivo models: from HNO cells and NPS biofluid. Immunoblot analysis confirmed the presence of seven out of eight well-characterized EV markers and the absence of GM130, a negative marker for EVs, in both EV groups. We observed an increased expression of ALIX in RSV-EVs compared to uninfected mock-EVs from HNOs and NPS samples, a similar result to what we previously observed in lower airway-derived EVs in response to RSV infection [26]. In the context of RSV infection, ALIX, ALG-2-interacting protein X, is a known protein recruited to the site of the viral assembly at the cell membrane facilitating the virus budding process [71]. Similar to our published finding of NPS-EVs [38], we found that RSV infection caused significant differences in the size and particle concentration of NPS-derived EVs. Indeed, viral budding and precursors of EV biogenesis share common pathways [72] that can influence the final expression of EV markers, as well as the size and particle number of EVs derived from two different systems (in vitro and ex vivo). However, changes in EV size following RSV infection may result from multiple interconnected mechanisms as changes in lipid within EVs. While ALIX enrichment in EVs from RSV groups could suggest selective recruitment during infection, which may not exclusively influence EV size through altered vesicle formation dynamics. Studies on other viral infections, such as HIV-1 [73] and Zika virus [74], have shown that viruses can manipulate EV biogenesis and content to enhance infectivity and modulate host responses. For example, lipidomic analyses have revealed that HIV-1 can be entrapped within EV aggregates, with these lipid-rich vesicles facilitating viral entry and replication in target cells. Similarly, Zika-infected mosquito cells release EVs enriched with viral proteins and RNA that not only infect naïve cells but also disrupt endothelial integrity and promote inflammation. These findings highlight how viral infections can reshape EV composition, particularly their lipid content, which in turn can influence EV size, cargo packaging, and downstream effects on recipient cells. It is plausible that RSV follows a similar paradigm, with virus-induced lipid remodeling altering EV structure and function during infection.

Then, we profiled the inflammatory and immune modulating proteins within EVs from human upper airway nose organoids and nasal secretions in RSV infected children using a novel methodology named PEA along with the known Bioplex platform. Previous studies have reported proteomic analyses of nasal secretion mainly in adult individuals with allergic rhinitis or nasal fluid from healthy subjects using proximity extension assays (PEA) [75,76]. Recent studies have begun to characterize age-related differences in cytokine responses to RSV in nasal epithelia cells. For instance, Alosio et al. demonstrated that RSV infection induces a distinct pro-inflammatory cytokine profile in nasal epithelial organoids derived from infants compared to adults, highlighting the developmental regulation of innate immune responses in early life [28]. Similarly, Wisgrill et al. profiled cytokine and chemokine release in primary nasal epithelial cells following viral stimulation with influenza A and RSV infections, and reported robust antiviral and inflammatory responses, further supporting the relevance of this model in studying airway immune responses [77]. A recent study by Woodall’s group has shown that pediatric nasal epithelial cells exhibit a distinct antiviral profile from adult cultures upon SARS-CoV-2 infection, characterized by the emergence of an inflammatory goblet cell subtype expressing high levels of interferon-stimulated genes and showing incomplete viral replication [78]. In contrast, older adult cultures display a shift toward basaloid-like cells that support viral spread and exhibit impaired epithelial repair mechanisms. These findings suggest that the nasal epithelium in children mounts a more robust and potentially protective early antiviral response, while aging may compromise these defense mechanisms and contribute to increased disease severity. These findings align with our observations and reinforce the importance of using nasal epithelial models in infants to understand RSV-driven inflammation in the context of age and disease severity. We acknowledge the potential for future research in this area and how it could enhance our understanding of the role of HNO in RSV infections in adults. However, no studies involving PEA technology have explored immune mediator proteins in the upper airway and in the context of viral infections in children. In this study, we aimed to focus primarily on specific inflammatory proteins associated with viral infections, along with a few samples of NPS derived EVs run for LC-MS/MS analysis. We plan for future experiments to increase the number of NPS derived EVs to examine the origin and circulating form of nasal derived EVs and expand the upper airway secretome study of proteins within EVs.

Our profiling of immune proteins via PEA technology in EVs from pediatric RSV-infected HNO cells and RSV-positive NPS samples revealed significant increases in several pro-inflammatory markers. We observed an enrichment of CCL2, MMP-1, MMP-10, IL-18R1, CXCL5, CCL20, CD40, CSF-1, CX3CL1, and TWEAK in RSV-EVs compared to Mock-EVs from HNOs. Alosio et al. reported that MMPs were detected in higher concentrations in pediatric-derived HNO-ALI cultures than in adult-derived HNO cells in response to RSV infection in the supernatant of HNO cultures ^28^. We were the first to observe MMPs concentrated in EVs of RSV-infected nasal organoids rather than in the supernatant as described in [28].

Additionally, we noted significant increases for uPA, TRAIL, ARNT, Flt3L, and CXCL6 in RSV-EVs compared to Mock-EVs from HNOs. Some of these proteins, such as uPA, IL-18R1, CXCL5, CCL20, and VEGFA were also confirmed in EVs from NPS biofluid of children positive for RSV. Previous studies found higher levels of the apoptotic TRAIL mediator in RSV-infected primary bronchial epithelial cells of children, and in primary small airway cells and A549 cells [79,80]. Our findings of concentrated TRAIL and TWEAK in EVs suggest that the two Tumor necrosis factors implicated in RSV-induced pathway could act through EV-mediated mechanisms during severe RSV disease.

Similar to our study, Krishnamachary’s group investigated the pro-inflammatory protein profile of blood-derived EVs in SARS-CoV-2-positive patients using PEA [37]. Interestingly, blood-derived EVs contained higher levels of EN-RAGE, TF, and IL-18R1, which correlated with disease severity and hospitalization duration. EVs from these patients also induced apoptosis in lung endothelial cells, further linking EVs to SARS-CoV-2 disease progression. Receptors for uPA, along with IL-18R1, Flt3L, and CCL2, were found in blood-derived EVs in response to SARS-CoV-2 [81]. It is known that uPA (Urokinase-Type Plasminogen Activator) and its receptor are associated with HIV [82], SARS-CoV-2, tissue remodeling and lung inflammation processes [83,84].

Our preliminary mass spectrometry data revealed that eosinophil cationic protein (ECP) is associated with EVs isolated from RSV-infected NPS. Del Pozo’s group identified ECP within EVs derived from activated blood eosinophils of patients with asthma [85]. Garofalo et al. demonstrated, for the first time, the presence of soluble, free-circulating ECP in the NPS biofluid of infants with RSV bronchiolitis and its association with the severity of clinical disease [86]. Previous studies by Garofalo’s team showed that RSV infection activated cytokine secretion by human eosinophils in an in vitro model [87]. We acknowledge that our mass spectrometry data and the other assays have certain limitations due to the small sample size, the timing of RSV sample collection, the variability in patient admission, age and sex -matched comparison not considered and different disease severities in response to RSV infection. To address these limitations, we plan to isolate EVs from a larger number of NPS samples and conduct a comprehensive LC-MS/MS study including the constraints of the study. The proteomic data obtained using PEA technology, combined with preliminary experiments on EVs from NPS samples analyzed by LC-MS/MS, have provided further insights into the cellular content and sources of EVs in NPS from infected patients.

Previously, we published that RSV infection significantly changed the piRNA profile in normal human small airway epithelia cells [25] and piRNAs were a major component of small noncoding RNAs of EVs from RSV-infected A549 cells [26]. This study of RNA sequencing analysis revealed that EVs isolated from NPS of infants positive for RSV infection displayed a different amount of piRNAs to the control (uninfected) EV samples. piRNAs are a type of small non coding RNA (sncRNAs) molecules regulating gene expression and silences transposons in animal cells [54]. Data in the field of RSV and piRNA profiling in nasal secretions or nasal derived EVs of infants with viral infections have not been investigated. Two studies demonstrated that miRNAs were detected in nasal secretions of children with episodes of rhinovirus and RSV infections. Nakstad and colleagues identified a distinct miRNA expression profile in the nasal mucosa of RSV-infected infants based on the disease severity of children with RSV infection [88]. Freishtat’s work suggested that children with episodes of rhinovirus or RSV bronchiolitis displayed a distinct nasal airway miRNA profile which differentially modulates the NFkB signaling pathway and downstream immune responses [89].

In our pilot experiment, we found that four piRNAs were upregulated, piR-32956, piR-33036, piR-33005, and piR-14633, and one downregulated, piR-33149 in EVs derived from RSV positive NPS samples. Target prediction analysis of the identified piRNAs in NPS derived EVs suggest that piR-32956 could potentially target Y RNA, a sncRNA type participating in a range of cellular processes including DNA replication, RNA quality control and cellular stress responses [56]. In the viral infection context, Y RNA can play a role in viral infections by acting as endogenous ligands for the RIG-I-like receptors (RLRs) of the immune system, essentially triggering an immune response when a virus infects a cell, particularly in the case of RNA viruses [90]. Recently, Y RNA was detected in EV from multiple cell lines and biofluids and maybe involved in a range of immune/inflammation related function [91] and with antiviral EV-mediated in response to influenza [92]. Similarly, piR-33036 target includes miRNA-30e-5p that was reported to have a critical role in innate immune responses during viral infections. The dysregulation of miRNA-30e-5p may contribute to the development and progression of autoimmune diseases like systemic lupus erythematosus [93].

These findings are biologically significant suggesting that cytokines, chemokines, and other proteins along with piRNAs are associated with EVs in response to RSV infection in pediatric upper airway, which serve as the primary entry point for respiratory pathogens. Due to the lipid structure of EVs, the proteins within them may be protected from cellular degradation while traveling from the upper to the lower airways, where the EV protein cargo could play a role in the response to lower airway viral infections ^12^. EVs generated in the upper airway mucosa are biologically active and could travel to the lung, modulating cell responses. Better understanding of the protein cargo of EVs released from upper airways following respiratory virus infection could provide insight into the regulation of viral-induced responses. In conclusion, these EVs could represent a novel translational approach for targeting previously undruggable pathways that do not respond to soluble, circulating cytokines.

## Figures and Tables

**Table 1:** Intensity-Based Absolute Quantification (iBAQ) and fold changes of cellular markers in NPS-EV.

Cellular Markers	Protein and Gene Names	Fold Change	*p* Value	iBAQ
B cells	Ig alpha-1 chain C region (IGHA1)	20.3	1.1 × 10^−2^	2.6 × 10^9^
	Ig kappa chain C region (IGKC)	7.2	3.9 × 10^−2^	1.6 × 10^9^
	Ig gamma-1 chain C region (IGHG1)	2.7	3.6 × 10^−2^	5.3 × 10^8^
	Ig alpha-2 chain C region (IGHA2)	53.5	1.2 × 10^−3^	3.6 × 10^7^
Eosinophils	Eosinophil cationic protein (RNASE3)	47.2	8.2 × 10^−3^	6.7 × 10^7^
Epithelial cells	Junction Plakoglobin (JUP)	−2.1	3.3 × 10^−1^	1.1 × 10^7^
	Desmoplakin [51]	−1.8	4.1 × 10^−1^	7.7 × 10^6^
	Moesin [52]	33.0	1.6 × 10^−4^	1.2 × 10^6^
Neutrophils	Neutrophil defensin 3 (DEFA3)	33.0	1.6 × 10^−4^	1.2 × 10^6^
	Neutrophil elastase [53]	57.1	1.3 × 10^−2^	5.6 × 10^10^
	Cathepsin G (CTSG)	1749.5	3.3 × 10^−4^	5.1 × 10^8^
	Azurocidin (AZU1)	207.8	2.4 × 10^−3^	3.2 × 10^8^
	Cathelicidin antimicrobial peptide (CAMP)	146.2	8.9 × 10^−5^	2.6 × 10^8^

**Table 2:** Fold change and predicted target genes of signifcanttly changed piRNAs in NPS derived EVs during the course of RSV infection.

piRNA	Fold Change (Log2)	Target Gene	Gene Name
piR-32956	4.08	Miscellaneous RNA	Y RNA
piR-33036	3.9	Miscellaneous RNA	MIR30E
piR-33005	3.58	Long non-coding RNA	LINC00623
piR-14633	3.47	-	-
piR-33149	−6.1	-	-

## Data Availability

The raw data supporting the conclusions of this article will be made available by the authors on request.
